# Therapeutic Advances in Pediatric Multiple Sclerosis

**DOI:** 10.3390/children12030259

**Published:** 2025-02-20

**Authors:** Rachel Walsh, Tanuja Chitnis

**Affiliations:** 1 Division of Child Neurology, Mass General Brigham Pediatric MS Center, Boston, MA 02114, USA; 2 Department of Neurology, Harvard Medical School, Boston, MA 02114, USA

**Keywords:** pediatric-onset multiple sclerosis (POMS), disease-modifying therapy, EDSS, PIRA, RAW, pediatric multiple sclerosis

## Abstract

Pediatric-onset multiple sclerosis (POMS) is a chronic, immune-mediated disorder that affects the central nervous system in children and adolescents. Approximately 3–10% of MS patients have an onset that occurs before the age of 18. The vast majority of pediatric MS cases are characterized by a relapsing-remitting course with a high burden of disease activity. Pediatric MS patients were historically treated off-label with varying degrees of success. With the approval of many new therapies for adult-onset MS, alternative treatments in pediatric MS have rapidly started to emerge. In this narrative review, we will discuss therapeutic advancements in pediatric multiple sclerosis, including the seminal trials of PARADIGMS, which evaluated fingolimod use in pediatric MS patients, CONNECT (dimethyl fumarate), TERIKIDS (teriflunomide), OPERETTA I (ocrelizumab), and LEMKIDS (alemtuzumab). We will also review the safety and efficacy of different monoclonal antibodies that are commonly prescribed for multiple sclerosis. We will then examine induction versus escalation treatment strategies and conclude with discussions on treatment considerations in POMS patients.

## 1. Introduction

Multiple sclerosis is an immune-mediated, inflammatory, and neurodegenerative condition [1]. It is characterized by demyelinating attacks on the central nervous system (CNS) that are disseminated in space and time [2]. Despite ongoing research, the underlying pathogenesis of MS has yet to be determined, and the etiology is thought to be multifactorial in nature, with both environmental and genetic contributions [3]. Multiple sclerosis can present at any age, and it is estimated that 3–10% of MS cases have an onset that occurs before the age of 18 [4]. Common clinical presentations include sensory and motor deficits, optic neuritis, brainstem syndromes, ataxia, and myelitis [2]. A diagnosis of pediatric MS can be made using the 2017 McDonald criteria, which defines dissemination in space (DIS) as one or more T2-hyperintense lesions in two or more areas of the CNS (periventricular, cortical, or juxtacortical, infratentorial brain regions and the spinal cord). Dissemination in time can be established by the presence of enhancing and non-enhancing lesions, oligoclonal bands specific to the CSF, or a new T2-hyperintense lesion on a follow-up MRI [5].

While pediatric and adult-onset MS share similar risk determinants and pathologic and clinical features, there are important differences to consider when treating pediatric MS patients [3,6,7]. Due to its inflammatory nature, pediatric-onset MS (POMS) commonly presents with a high number of demyelinating lesions and disease activity. Annualized relapse rates are more than twice as high in POMS compared to adult-onset MS (AOMS) during the early stages of the disease [8,9]. POMS almost exclusively presents as a relapse-remitting disease, and pediatric patients accrue disability at a slower rate compared to adult patients [3,10,11]. Despite this distinction, the shift to a secondary progressive phenotype occurs at a chronological age, on average, ten years younger than those with AOMS [12].

The ultimate goal in the treatment of MS is to delay disability milestones for as long as possible. There are two processes by which MS patients accumulate disability: clinical relapse-associated worsening (RAW) and progression independent of relapse activity (PIRA). Understanding these mechanisms and how clinical relapses influence disease progression over time is an active area of research [13]. As RAW is the dominant driver of disability in pediatric-onset RRMS, current disease-modifying therapies were designed to target focal inflammation and prevent clinical relapses [13]. This paper aims to review therapeutic advancements in pediatric multiple sclerosis over the past three decades, highlighting the different approaches to treatment and promising developments ahead.

## 2. Methods

A comprehensive literature search was conducted using the PubMed database for this narrative review. Articles pertaining to pediatric multiple sclerosis, multiple sclerosis, and disease-modifying therapies were searched up until 1 December 2024. Article selection was performed at the discretion of the authors and included abstracts, textbook chapters, clinical and narrative reviews, case series, and phase II and III clinical trials.

## 3. Disease-Modifying Therapy

Disease-modifying therapies for multiple sclerosis are designed to reduce clinical relapse rates, slow the accumulation of disability, and decrease MRI disease activity [14]. Pediatric multiple sclerosis is treated with a variety of disease-modifying therapies, including interferons, glatiramer acetate, fumarates, teriflunomide, anti-CD52 and CD20 monoclonal antibodies, natalizumab, and fingolimod.

## 4. Immunomodulators

Throughout the 1990s, several phase III clinical trials were conducted that led to the approval and worldwide use of both glatiramer acetate and interferon beta for the treatment of adult-onset multiple sclerosis [15]. As patients younger than 18 years old were not included, providers largely extrapolated from these results to treat POMS patients and published investigator-initiated observational studies [16,17,18,19,20,21,22,23,24].

## 5. Interferon Beta

Type I interferons, which include interferon alpha and interferon beta, affect the immune system through several different mechanisms [25]. They promote an anti-inflammatory state through their actions on dendritic cells, T and B lymphocytes, and NK and T regulatory cells [26]. Interferons were initially investigated as antiviral treatments against several infectious diseases in the 1980s [15]. As many started to consider a viral trigger in the pathogenesis of MS, scientists investigated interferon beta as a potential treatment option [15]. Several studies confirmed its clinical benefit, and in 1993, IFN-β-1b (Betaseron/Betaferon) became the first DMT approved by the FDA for MS in individuals older than 18 years, shortly followed by a different formulation, IFN-β-1a, delivered via IM injection (Avonex) and subcutaneous injection (Rebif) [27].

In 2001, two case series (*n* = 9, *n* = 13) assessing the use of IFN-β-1a (Avonex) in pediatric patients found that the treatment was safe in both cohorts, with the most reported side effects being flu-like symptoms, injection-site reactions, and headaches [16,17]. In an effort to assess the efficacy of IFN-β-1a (Rebif) in POMS, Pohl et al. analyzed a larger sample of pediatric patients (*n* = 51) treated for a mean duration of 1.8 years [18]. The annualized relapse rate in this study decreased from 1.9 to 0.8. In a prospective randomized trial published in 2006, sixteen pediatric patients with RRMS were divided into two treatment groups: those treated with IFN-β-1a (Avonex) and no disease-modifying therapy. After four years of follow-up, the group of patients who did not receive therapy experienced twice as many relapses (*n* = 35) compared to those who did receive therapy (*n* = 19) [19]. Banwell et al. investigated the use of IFN-β-1b (Betaseron/Betaferon) in a cohort of 43 children and adolescents with MS and similarly observed a reduction in ARR of 50% [20]. A retrospective phase IV study (REPLAY) published in 2013 reviewed the safety and tolerability of IFN-β-1a (Rebif) in a large, multinational pediatric MS population (*n* = 298) [21]. Nonserious medical events were common, occurring in 60% of patients, with transaminitis found in approximately 30% of patients [21]. The annualized relapse rate decreased from 1.79 to 0.47 during a mean treatment period of 2.11 years. Overall, this study reaffirmed that IFN-β is efficacious in the pediatric population, generally well tolerated, and associated with a reduction in relapse rates [21].

## 6. Glatiramer Acetate

Glatiramer acetate (GA) is a synthetic mixture of four amino acid copolymers developed to resemble myelin basic protein [28]. In addition to competing with and displacing myelin antigens on antigen-presenting cells, it has been proposed that GA also stimulates the release of anti-inflammatory cytokines by T cells, suppresses proinflammatory Th1 cells, and reduces the activation of monocytes [29]. When it reduced the severity of autoimmune disease in mouse models, investigators explored its potential as a treatment for MS [29]. Following a phase III randomized controlled trial that demonstrated a 30% reduction in relapse rate, GA was approved by the FDA in 1996 for the treatment of MS in the adult population [29].

Kornek et al. published one of the first case series (*n* = 7) investigating GA treatment in pediatric MS patients in 2003 [22]. The patients were followed for 24 months, and mild side effects were reported. In 2005, an Italian registry evaluated a large series of pediatric MS patients who were treated with either GA or IFN-β. In the nine patients treated with GA, the mean annualized relapse rate decreased from 2.8 to 0.25 [23]. A Brazilian cohort (*n* = 15) found similar results with a reported annualized relapse rate reduction from 2.5 to 0.53 [24].

In 2012, the International Pediatric MS Study Group (IPMSSG) presented a consensus statement and recommended that all pediatric MS patients be started on either beta-interferon or glatiramer acetate as the first-line therapy [30]. In the event that a patient had an inadequate treatment response, the IPMSSG recommended switching between first-line therapies or to a second-line agent. The decision to switch to a different therapy was left to the discretion of the individual provider in collaboration with the patient and their family [30].

While injectable immunomodulators are considered to have low safety risks, they are often ineffective in preventing clinical relapses [31]. A retrospective analysis of a large pediatric MS cohort found that among those receiving GA (*n* = 53) and IFN-β (*n* = 200), 20% and 30%, respectively, were switched to a different therapy after a mean of 1.3 years due to refractory disease [32]. Given this, combined with the concurrent approval of many new therapies for adult-onset MS, reports of different therapies and treatment strategies in pediatric MS have rapidly started to emerge.

## 7. Alkylating Agents

### Cyclophosphamide

Cyclophosphamide (Cytoxan) has been prescribed as both an antineoplastic and immunosuppressant therapy for many decades [33]. It acts as an alkylating agent and interferes with DNA synthesis, thereby reducing the number of circulating immune cells [33,34]. Many open-label studies have demonstrated its efficacy as a second-line agent in the adult MS population, particularly in patients with active and inflammatory disease [26,34]. Makhani et al. evaluated the use of three different cyclophosphamide dosing regimens (induction therapy alone, induction therapy with pulse maintenance therapy, or pulse maintenance therapy alone) in seventeen pediatric MS patients [35]. In the fourteen patients, who received pulse maintenance therapy, the mean ARR decreased from 3.8 in the year prior to starting cyclophosphamide treatment to 1.1 during the first year of therapy [35]. Following cyclophosphamide discontinuation, nine patients (53%) presented with a clinical relapse over a mean follow-up period of 2.7 years [35]. Common side effects reported included nausea, vomiting, infection, anemia, and alopecia [35]. Two patients developed serious complications: infertility, as confirmed by fertility tests, and transitional cell carcinoma of the bladder [35]. The risk of acquiring these severe side effects appears to increase with drug exposure [34]. The results of this study suggest that an induction course of cyclophosphamide could be considered in highly inflammatory cases that are refractory to traditional first-line treatments [35].

## 8. S1P Receptor Modulators

### Fingolimod

For lymphocytes to enter the systemic circulation, S1P, a signaling sphingolipid, must activate its cell surface receptors [36]. Fingolimod (Gilenya) acts as a functional antagonist and binds to S1P receptors, triggering their internalization and degradation. This, in effect, retains lymphocytes in secondary lymphoid organs [26]. Based on the results from PARADIGMS, a phase III, double-blind, randomized clinical trial, fingolimod is the only medication approved by both the FDA and EU to treat MS in pediatric patients [12]. The study enrolled 215 pediatric patients aged 10–17 years at eighty centers in twenty-five countries [12]. Patients were randomly assigned at a 1:1 ratio to receive either fingolimod or intramuscular IFN-β-1a. The results were significant for an 82% relapse reduction in the fingolimod-treated patients compared to the IFN-β-1a-treated patients. The time to first relapse was also considerably delayed in the fingolimod treatment arm, with 85.7% of patients free of confirmed relapses at month 24. Serious adverse events occurred in 18 patients (16.8%) treated with fingolimod, which included seizures, leukopenia, and a single case of Mobitz type I second-degree AV block. Fingolimod was overall well tolerated, and fewer patients in this treatment group discontinued the trial prematurely. A 5-year extension trial evaluating the safety and efficacy of fingolimod is ongoing [12].

## 9. Monoclonal Antibodies

### Natalizumab

Natalizumab (Tysabri) is a humanized monoclonal antibody designed to target the α4 subunit of α4β1integrin on leukocytes, thereby inhibiting their ability to bind to cell adhesion molecules and migrate across the blood–brain barrier [37]. Natalizumab has been shown to be a highly effective therapy in pediatric MS in several observational studies. In an Italian report of 101 patients, the ARR decreased from 2.3 ± 1.0 in the year prior to natalizumab initiation to 0.1 ± 0.3 [38]. Mild-to-moderate side effects, including infections, hypersensitivity reactions, and hematologic abnormalities, were observed in 36% of patients, none of which required the discontinuation of treatment. Comparable efficacy and safety results were reported in a case series (*n* = 32) from the Kuwait national MS registry [39]. In another cohort of Italian patients (*n* = 20) treated with natalizumab as the first-line therapy, no clinical relapses were observed in the 24 months following treatment initiation [40]. More recently, a multicenter cohort study from the US registry examined 151 pediatric patients treated with natalizumab and reported a decrease in the relapse rate from 0.96 in the year prior to initiating treatment to 0.13 while treatment was ongoing [41].

Despite its efficacy, progressive multifocal leukoencephalopathy (PML) is a feared complication of natalizumab and is associated with significant mortality. PML is an opportunistic infection triggered by the reactivation of the JC virus [42]. The risk of developing PML is increased in patients with a positive JCV antibody status prior to the use of immunosuppressants or treatment with natalizumab for more than 24 months [43]. In a recent study of an Italian pediatric MS cohort, approximately one-half of patients discontinued natalizumab treatment at follow-up, with the majority stopping due to their JCV-positive status [44]. Notably, despite initiation with another agent, over half of these patients experienced disease reactivation [44]. While natalizumab is safe for administration in pediatric patients, close surveillance during and after the treatment period is warranted [45].

## 10. Alemtuzumab

Alemtuzumab (Lemtrada) is a humanized monoclonal antibody that targets the surface antigen CD52 and depletes circulating B and T lymphocytes by cytolysis [36]. Following a phase III trial that demonstrated reduced relapse rates and improved MRI outcomes, alemtuzumab was approved for the treatment of adult MS [46]. There were several adverse events reported, including the development of autoimmune conditions, opportunistic infections, and acute cerebrovascular disorders [46]. There were no serious adverse events or definite relapses reported in two small case series evaluating alemtuzumab use in pediatric patients [47,48]. The LemKids study, a phase III clinical trial designed to assess the safety, efficacy, and tolerability of alemtuzumab in pediatric MS patients, was prematurely terminated due to low enrollment [49]. Recruitment was temporarily suspended in 2019 when the European Commission initiated a safety review and briefly restricted the use of alemtuzumab in MS patients. Based on the limited data collected, treatment with alemtuzumab in 11 children with a mean age of 14.5 years was associated with fewer new or enlarging T2 lesions compared with prior DMT, and there were no severe adverse events reported [49].

## 11. Ocrelizumab and Rituximab

Rituximab (Rituxan) and ocrelizumab (Ocrevus) are both monoclonal antibodies that bind to the antigen CD20 and interfere with B-cell activation and differentiation [50]. Following successful phase III trials in 2015, ocrelizumab, a humanized anti-CD20 monoclonal antibody, was approved for the treatment of MS in adult patients in both Europe and the US [43]. Many observational studies report favorable risk–benefit ratios for both rituximab and ocrelizumab in the pediatric population [41,51,52,53,54]. In a multicenter European cohort, the annualized relapse rate decreased from 0.6 to 0.03 in patients (*n* = 61) treated with rituximab [55]. The adverse events reported included infusion-related complications (48%), infection (20%), hypogammaglobulinemia (17%), and lymphopenia (7%) [55]. OPERETTA I, a phase II clinical trial, was designed to evaluate the safety, pharmacokinetic, and pharmacodynamic effects of ocrelizumab in pediatric MS patients [56]. There were no clinical relapses reported during the 2-year study [56]. The safety profile was similar to those described in adult MS trials, and the most frequent adverse events reported were infusion-related reactions and headaches [56]. Based on the results of this study, a dosing regimen was selected for the ongoing Phase III OPERETTA 2 trial (NCT05123703) [57].

## 12. Pyrimidine Synthesis Inhibitor

### Teriflunomide

Teriflunomide (Aubagio) inhibits dihydroorotate dehydrogenase, an enzyme in the pyrimidine synthesis pathway, and interferes with cell replication in T and B lymphocytes [58,59]. It was approved to treat MS in adult patients by the FDA in 2012 and the EU in 2013 [36]. Between 2014 and 2019, 166 pediatric patients from 22 different countries enrolled in TERIKIDS, a phase III, double-blind, placebo-controlled trial evaluating the safety and efficacy of teriflunomide in POMS [60]. Patients were randomly assigned in a 2:1 ratio to receive either teriflunomide or a placebo for up to 96 weeks [60]. Teriflunomide reduced the amount of new or enlarging T2 and contrast-enhancing T1 lesions by 55% and 75%, respectively [60]. However, there was not a statistically significant difference in the time to first confirmed relapse between the treatment and placebo group [60]. This is likely attributable to the fact that 26% of patients in the placebo arm switched to the open-label extension due to high MRI activity, resulting in a smaller, more clinically stable comparison group [60].

To better assess the long-term safety and efficacy of teriflunomide, an additional 96-week extension trial further followed the patients who completed the double-blind period or switched to the treatment arm early [61]. Two-thirds of patients receiving teriflunomide reported mild-to-moderate infections versus 46% in the placebo group. Additional adverse events described more frequently in the teriflunomide group included alopecia (21%), paresthesia (11%), abdominal pain (11%), increased serum CK (6%), pancreatic disorders (6%), and elevated liver enzymes (4.6%) [60]. Following the publication of TERIKIDS, the European Commission approved teriflunomide for the treatment of MS in pediatric patients.

## 13. Fumarates

### Dimethyl Fumarate (DMF)

DMF (Tecfidera) is a fumaric acid ester that affects the nervous and immune systems in a variety of ways [58]. It activates Nrf-2-dependent and independent pathways, altering the composition of immune cells and shifting the immune response towards a more anti-inflammatory state [62]. Three pivotal studies, FOCUS, CONNECTED, and CONNECT, examined the use of DMF in pediatric MS patients [63,64,65]. CONNECT, a 96-week phase III randomized, open-label trial, is the most recent study to compare the effectiveness of DMF with IM interferon-β-1a [65]. A total of 103 patients completed the study and were included in the primary end point analysis [65]. Although the study lacked statistical power to significantly compare the two treatment groups, the percentage of patients without new or enlarging T2 lesions was considerably higher in those receiving DMF (16.1%) compared to IFN (4.9%) [65].

Adverse events more commonly reported in the DMF treatment group (*n* = 78) included GI intolerability (74.4%), infection (52.6%), abdominal pain (41%), and flushing (38.5%) [65]. Two patients developed lymphopenia, one of which was severe and led to the discontinuation of treatment [65]. The safety findings reported in the CONNECT trial are largely consistent with the adverse events reported in prior pediatric and adult MS studies [63,64,65]. While GI and flushing side effects are common, they are often mild in severity and reduce in frequency over the first few months of treatment [66]. DMF is approved in the EU for the treatment of pediatric MS in patients aged 13 years and older.

## 14. DMT Landscape over Time

Several observational studies have evaluated the effectiveness of initial disease-modifying therapies [67,68]. In one US report, patients who started on high-efficacy treatments were less likely to have a clinical or radiographic relapse compared to those started on injectable therapies [67]. Similar results were reported in a UK study where relapses occurred in 59.6% (53/89) of patients on injectable therapies versus 15% (8/54) of patients on newer DMTs [69]. Additionally, in a pediatric French cohort, the mean ARR for patients treated with fingolimod, teriflunomide, DMF, or natalizumab was significantly lower than for those treated with interferon [70]. In 2022, Graves et al. published a meta-analysis of 19 studies conducted on participants from around the world and found that the ARR was significantly higher in patients treated with IFN than those treated with fingolimod or natalizumab [68]. Similarly, a large multinational retrospective study published in 2024 analyzed 1218 POMS patients treated with either fingolimod, natalizumab, or an injectable therapy [71]. At the five-year follow-up, the percentage of patients that remained relapse-free was highest for those in the natalizumab treatment group (90%), followed by fingolimod (72%) and injectable DMTs (36%) [71].

Owing to their demonstrated effectiveness, a growing number of pediatric MS patients are prescribed high-efficacy disease-modifying therapies [72]. In an international survey completed by 66 members of the IPMSSG, 44% of providers stated that they had prescribed a high-efficacy DMT to pediatric patients [73]. In the US, a multicohort observational study revealed that compared to 2008, when 100% of patients older than 12 years started treatment with IFN or GA, only 48% were prescribed these medications in 2016 [31]. Knowing that pediatric MS is highly inflammatory and that early high-efficacy therapy is associated with a reduction in disease progression in the adult population [74], an increasing number of providers prescribe highly effective therapies at disease onset [75]. An induction strategy is supported by the results of a recent observational study, which reported patients who switched to high-efficacy therapies within two years are more likely to remain relapse-free at follow-up compared to those started on a higher efficacy therapy more than two years later [76]. Further investigation is needed, however, to better understand the risk–benefit ratio of long-term exposure to high-efficacy DMTs in the pediatric population.

Although there are many studies that confirm the fact that oral and infusion MS therapies are highly effective in reducing disease activity, there is no recent consensus available to guide initial treatment selection [67]. In a recent multicenter French report, moderately effective therapies (azathioprine, cyclophosphamide, DMF, GA, IFN, MTX, mycophenolate mofetil, and teriflunomide) were compared to highly effective therapies (alemtuzumab, fingolimod, mitoxantrone, natalizumab, ocrelizumab, ofatumumab, and rituximab) [74]. There was a greater reduction in the annualized relapse rate in those treated with higher efficacy therapies [74]. Similar findings were reported in a US cohort that evaluated the effectiveness of injectable, oral, and infusion therapies [67]. When compared to injectable treatments, infusion therapies (natalizumab, rituximab, and ocrelizumab) seemed to be more effective than oral therapies (DMF, fingolimod, and teriflunomide) in lowering disease burden [67]. These studies underscore the importance of further prospective research efforts to identify optimal treatment strategies in pediatric-onset MS.

## 15. Safety Considerations

There are no large differences in DMT safety parameters between pediatric and adult MS patients [41]. Short-term side effects of disease-modifying therapies in children are comparable to those reported in adult trials [31]. The risk of contracting a SARS-CoV-2 infection impacted the treatment decisions of many providers at the beginning of the pandemic [73]. In one US cohort evaluating the risks in patients using disease-modifying therapy, 12.3% (9/73) of COVID-19-positive patients were hospitalized [77]. Of the hospitalized patients, 89% (8/9) were prescribed B-cell-depleting therapies, and all survived their hospital course [77]. As new information continues to emerge, the benefits of continuing MS treatment in pediatric patients generally seem to outweigh the risks of discontinuing therapy [75].

Given that disease-modifying therapies can increase the risk of infections, vaccine status is an important consideration. All primary vaccines should be administered prior to starting disease-modifying therapy, and the confirmation of immunity against varicella is recommended. Live vaccines should not be administered unless first discussed with the treating neurologist [75].

## 16. Selecting Disease-Modifying Therapy for Patients

At present, glatiramer acetate, IFN-β, fingolimod, teriflunomide, and dimethyl fumarate are approved by the EMA for use as treatment in pediatric patients with RRMS. Fingolimod continues to be the only DMT for pediatric MS that is FDA-approved. Providers around the world often prescribe medications approved in adult-onset MS off-label to treat their pediatric patients. These therapies, most frequently used in a recent multicenter US study, are DMF (*n* = 168), natalizumab (*n* = 151), rituximab (*n* = 166), fingolimod (*n* = 96), and ocrelizumab (*n* = 37). In a survey completed by members of the IPMSSG in 2021, the preferred first choice of DMTs were IFN-β and fingolimod, followed by rituximab, natalizumab and GA, followed by DMF and ocrelizumab [73]. Not starting a particular DMT due to limited access was reported for anti-CD20 therapies, teriflunomide, and DMF [73]. Therapeutic decisions are currently made on an individual basis and influenced by a variety of factors, including the patient’s age, disease features, comorbidities, insurance authorization, and method of delivery. In each case, providers must consider which medication is most appropriate and discuss its risks and benefits with the patient and their family. Following initial treatment selection, close monitoring for side effects and breakthrough disease is strongly recommended.

## 17. Wellness as a Treatment

Lifestyle modifications play an important role in mitigating the determinants of disease risk. Adolescent obesity is a known risk factor for pediatric and adult-onset MS. It is recommended that all patients engage in physical activity and incorporate fresh whole foods into their diet as much as possible. Greater physical activity has been associated with a lower disease burden and fewer fatigue symptoms, and high-energy intake from saturated fats may increase the likelihood of relapse [78,79]. Secondhand tobacco exposure is also an independent risk factor for central nervous system demyelination and should be avoided in all children [80]. While the impact of vitamin D supplementation on pediatric MS prognosis is unknown, genetic polymorphisms in the vitamin D pathway have been associated with serum levels and relapse rates in MS [81]. It is, therefore, generally recommended that patients reach target levels above 30 ng/mL. It is essential that providers discuss these risk factors with their patients and set tenable goals to review at each visit [82].

## 18. Conclusions and Next Steps

The advancements in multiple sclerosis treatments over the past two decades have been significant. New and emerging medium- and high-efficacy therapies, including dimethyl fumarate, teriflunomide, ocrelizumab, fingolimod, and natalizumab, are superior in preventing clinical relapses in pediatric-onset MS and show promise in decreasing the risk of disability over time [83]. More robust clinical studies evaluating the long-term safety and efficacy of DMTs in children are needed to guide evidence-based practice. There are currently two active phase III clinical trials underway: NEOS 3, a double-blind non-inferiority study comparing the efficacy of ofatumumab and siponimod versus fingolimod in pediatric MS patients, and OPERETTA 2, a randomized, double-blind study evaluating the safety and efficacy of ocrelizumab in comparison with fingolimod [57,84]. Given that there are often feasibility constraints and limitations due to small sample sizes in pediatric MS, international collaboration is crucial, and innovative trial designs that demonstrate effectiveness should be considered for regulatory approval [68].

While many studies have concluded that high-efficacy disease-modifying therapies are effective and well tolerated in the pediatric population, there is limited data on the impact of these treatments on subclinical and progressive MS disease activity, including the accrual of cortical lesions and brain atrophy over time. The ideal duration of high-efficacy disease-modifying treatment in pediatric patients is also not known. Determining additional objective clinical and radiographic markers is critical to assist in management decisions. Advanced diagnostic testing, including higher resolution magnetic resonance imaging and wearable sensors, may help to prognose the severity of the disease in the future. Future clinical trials need to address the potential biomarkers of active disease, optimal treatment intervals, and long-term physical and neurocognitive outcomes in pediatric MS patients.
